# Pulmonary Delivery of Fenretinide: A Possible Adjuvant Treatment in COVID-19

**DOI:** 10.3390/ijms21113812

**Published:** 2020-05-27

**Authors:** Isabella Orienti, Giovanna Angela Gentilomi, Giovanna Farruggia

**Affiliations:** 1 Department of Pharmacy and Biotechnology, Alma Mater Studiorum-University of Bologna, Via S. Donato 19/2, 40127 Bologna, Italy; giovanna.farruggia@unibo.it; 2 Department of Pharmacy and Biotechnology, Alma Mater Studiorum-University of Bologna, Via Massarenti 9, 40138 Bologna, Italy; giovanna.gentilomi@unibo.it; 3 Unit of Microbiology, Alma Mater Studiorum-University of Bologna, S. Orsola-Malpighi Hospital, 40138 Bologna, Italy; 4 Biostructures and Biosystems National Institute (BBNI), 00136 Roma, Italy

**Keywords:** COVID-19, fenretinide, anti-inflammatory, antiviral environment, pulmonary delivery, adjuvant treatment

## Abstract

At present, there is no vaccine or effective standard treatment for severe acute respiratory syndrome coronavirus-2 (SARS-CoV-2) infection (or coronavirus disease-19 (COVID-19)), which frequently leads to lethal pulmonary inflammatory responses. COVID-19 pathology is characterized by extreme inflammation and amplified immune response with activation of a cytokine storm. A subsequent progression to acute lung injury (ALI) or acute respiratory distress syndrome (ARDS) can take place, which is often followed by death. The causes of these strong inflammatory responses in SARS-CoV-2 infection are still unknown. As uncontrolled pulmonary inflammation is likely the main cause of death in SARS-CoV-2 infection, anti-inflammatory therapeutic interventions are particularly important. Fenretinide N-(4-hydroxyphenyl) retinamide is a bioactive molecule characterized by poly-pharmacological properties and a low toxicity profile. Fenretinide is endowed with antitumor, anti-inflammatory, antiviral, and immunomodulating properties other than efficacy in obesity/diabetic pathologies. Its anti-inflammatory and antiviral activities, in particular, could likely have utility in multimodal therapies for the treatment of ALI/ARDS in COVID-19 patients. Moreover, fenretinide administration by pulmonary delivery systems could further increase its therapeutic value by carrying high drug concentrations to the lungs and triggering a rapid onset of activity. This is particularly important in SARS-CoV-2 infection, where only a narrow time window exists for therapeutic intervention.

## 1. Introduction

Coronaviruses (CoVs) are RNA viruses. They may infect both humans and animals, leading to lethal and contagious diseases, such as severe acute respiratory syndrome coronavirus (SARS-CoV) and Middle East respiratory syndrome coronavirus (MERS-CoV). The recent severe acute respiratory syndrome coronavirus-2 (SARS-CoV-2) (or coronavirus disease-19 (COVID-19)) is characterized by high genetic homology with SARS-CoV and MERS-CoV [1], sharing with them 79.0% and 51.8% nucleotide identity, respectively. Several studies have shown that SARS-CoV predominantly infects airway and alveolar epithelial cells, vascular endothelial cells, and macrophages. SARS-CoV-2 and SARS-CoV use the same receptor, angiotensin-converting enzyme 2 (ACE2), for infection, indicating that the same cell types are targeted and infected [2,3].

In SARS-CoV-2 infection, the early onset of a rapid viral replication may cause extensive apoptosis of epithelial and endothelial cells and vascular leakage. This frequently leads to acute lung injury and lethal inflammatory responses [4]. Although antiviral drugs, glucocorticoids, and mechanical ventilation have been used, there is no specific treatment for COVID-19 at the moment [5]. Therefore, in the absence of a standard therapeutic intervention, the urgent treatment of pulmonary inflammation to prevent acute lung injury is needed. Pulmonary formulations of anti-inflammatory drugs could represent a good option in combination with systemic antiviral drugs or glucocorticoids. In pulmonary administration, the drugs are directly carried to the airway and alveolar epithelia in high concentration, providing rapid onset of the therapeutic response with prompt relief of lung occlusion and respiratory distress symptoms. Nevertheless, the drugs used by pulmonary administration in COVID-19 should be characterized by low toxicity to avoid injury to the airway and alveolar epithelia already damaged by the viral infection. Fenretinide—(*N*-(4-hydroxyphenyl) retinamide)—is a semisynthetic derivative of all-*trans*-retinoic acid. It is endowed with many pharmacological features, including anti-inflammatory and antiviral activities, prevention of obesity and type-2 diabetes [6], and the well-known antitumor activity on a wide range of tumors [7,8,9,10,11,12,13,14,15,16]. Moreover, its low toxicity profile has been proved in many clinical trials, also in long term treatments [12,17,18]. Therefore, due to its poly-pharmacology, fenretinide administration by pulmonary formulations may be expected to be protective against acute lung injury (ALI)/ acute respiratory distress syndrome (ARDS) caused by SARS-CoV infection and could represent a useful tool in a multimodal therapy aimed at establishing a rapid anti-inflammatory and antiviral effect.

## 2. Inflammation Caused by SARS-CoV

Pulmonary hyper inflammation is a common feature in COVID-19 patients. When SARS-CoV is inhaled and infects epithelial cells in the lungs, an immune response is activated by the local dendritic cells that phagocytose the virus and circulate to the regional lymph nodes where they present antigens to T cells. Activated T cells migrate to the lungs, where they mount the immune response. 

**Figure 1:**
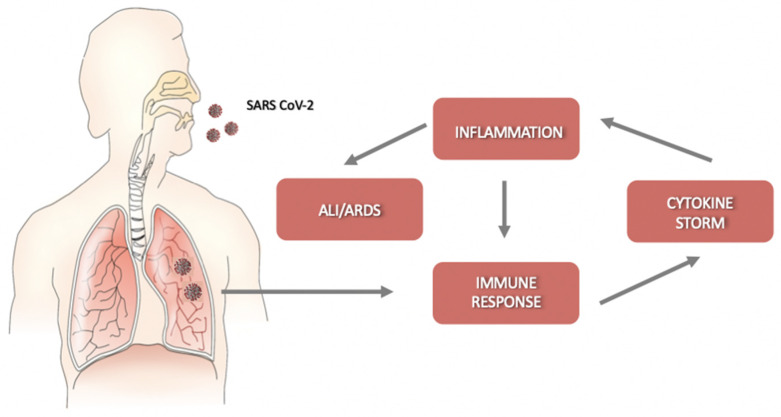
Schematic representation of the progression of coronavirus disease-19 (COVID-19). After an incubation period, severe acute respiratory syndrome coronavirus-2 (SARS-CoV-2) starts a rapid replication in the lung airway and alveolar epithelial cells. This triggers an immune response with cytokine production, excessive inflammation, and further amplification of the immune response that triggers the cytokine storm. Acute lung injury (ALI)/ acute respiratory distress syndrome (ARDS) may arise with dire consequences.

Helper T cells secrete cytokines that regulate and assist the immune response; cytotoxic CD8^+^ T cells (CTLs) recognize and kill the infected cells. They release pro-inflammatory cytokines, perforins, and granzymes to induce programmed cell death [19]. However, the cytokines produced by CTLs can damage uninfected as well as virus-infected cells, and their massive production may cause detrimental effects, leading to acute lung injury. SARS-CoV-2 infection is characterized by a rapid viral replication that triggers an amplified immune response with the activation of a cytokine storm. The consequent massive inflammation and increase in vascular permeability induce an abnormal accumulation of neutrophils, macrophages, inflammatory monocytes, and lymphocytes in the lung alveoli, further increasing cytokine production [20]. In this condition, the regulation of the immune response is lost, and the cytokine storm is further activated. Without therapeutic intervention, the development of ALI/ARDS and permanent alterations in lung functions may result in dire consequences [4] (Figure 1). Moreover, the circulation of cytokines to other organs can lead to multi-organ damage.

## 3. Anti-Inflammatory Activity of Fenretinide

Fenretinide is the most investigated retinoid due to a significant antitumor activity on a wide range of tumor types [7,8,9,10,11,12,13,14,15,16] combined with a favorable toxicological profile [12,17,18]. Besides its antitumor activity, multimodal interactions with several biological mechanisms endow fenretinide with other pharmacological properties, including anti-inflammatory activity, antiviral activity, and prevention of obesity and type-2 diabetes [6]. The anti-inflammatory activity of fenretinide has been largely proved, and its mechanism of action has been characterized in different pathological conditions. In inflamed tissues, where the macrophages are characterized by a significantly higher ratio of arachidonic acid (AA) vs. docosahexaenoic acid (DHA) than in normal tissues, fenretinide induced normalization of the AA/DHA ratio, thus inhibiting phosphorylation of ERK1/2 and decreasing the expression of inflammatory cytokines [21]. Moreover, fenretinide’s ability to downregulate the production of AA and increase the levels of DHA provided attenuation of inflammation in cystic fibrosis [22], osteoporosis [23], and in a model of spinal cord injury [24]. In a mouse model of allergic asthma, fenretinide inhibited Lypopolysaccaride (LPS)-induced expression of various Th1 and Th2 cytokines, including Ccl5, Ccl7, Ccl11, Cxcl1, Cxcl2, Cxcl9, Cxcl10, Interleukin (IL)-6, TNF-α, and iNOS [25]. In LPS-exposed mouse brain microvascular endothelial cells (bEnd.3 cells), fenretinide showed inhibitory effects on the release of pro-inflammatory cytokines (IL-1β, MCP1, iNOS, and TNF-α). Fenretinide may inhibit NF-κB signaling by reducing its nuclear translocation via downregulation of IKKβ and IκBα phosphorylation [26]. This hampers the secretion of inflammatory modulators that is closely associated with the activation of NF-κB signaling. In mouse monocyte/macrophage cells infected with *Aggregatibacter actinomycetemcomitans*, fenretinide suppressed JAK-STAT, PI3K-Akt, PKC, and downstream NF-κB signaling pathways, therefore attenuating IL-1β, IL-6, and PGE2 proinflammatory cytokine expression [27]. 

Fenretinide has been demonstrated to induce Reactive Oxygen Species (ROS) generation and oxidative stress in tumor cells as a part of its cytotoxic mechanism [28,29]. In contrast, it has not increased ROS in normal cells, such as fibroblasts, peripheral blood mononuclear cells [29], and normal and activated peripheral lymphocytes [30]. Therefore, its anti-inflammatory activity in non-tumor tissues is not counterbalanced by the ROS-inducing activity, and no detrimental effect can be expected when fenretinide is applied to non-tumor tissues.

## 4. Antiviral Activity of Fenretinide 

The antiviral activity of fenretinide has been proved in different virus types. In a screening study using a Library of Pharmacologically Active Compounds (LOPAC) of bioactive compounds against the respiratory syncytial virus (RSV), fenretinide was identified as a specific blocker [31]. In the dengue virus (DENV), fenretinide has shown high activity both in vitro and in vivo [32]. Its mechanism of action includes inhibition of DENV non-structural protein 5 (NS5) recognition by host nuclear import proteins importin-α/β1; upregulation of transcripts representing the protein kinase R-like endoplasmic reticulum kinase (PERK) arm of the unfolded protein response (UPR); phosphorylation of eukaryotic translation initiation factor 2α (eIF2α) with consequent translation attenuation and induction of an antiviral state [33]. Fenretinide inhibited the steady-state accumulation of viral genomic RNA and reduced viremia when orally administered in a murine model of DENV infection. The molecular target responsible for this antiviral activity was distinct from other known inhibitors of DENV and appeared to affect other members of the Flaviviridae, including the West Nile, Modoc, and hepatitis C viruses [34]. Fenretinide inhibited the zika virus (ZIKV) in mammalian cell culture and reduced both viremia and brain viral burden in a murine model. This antiviral activity was correlated with a significant decrease in the abundance of ZIKV RNA. It was found that fenretinide reduced RNA synthesis without direct inhibition of the viral polymerase or chemical destabilization of membrane-associated replication complexes, rather, fenretinide acted via an indirect mechanism mediated by a host factor [35]. In both ZIKV and DENV, fenretinide inhibited the non-structural protein 5 (NS5), which contributed to pathogenesis, by antagonism with the production of interferon-I (IFN-I) [36]. As it is well-known, IFN-I promotes an early protective host mechanism against viral infection, by driving an antiviral state in non-immune cells and activating antiviral immune responses. Therefore, the reestablishment of IFN-I production by fenretinide can block the early onset of viral infection [37]. 

The delayed release of IFN-I is well known in SARS-CoV and MERS-CoV infections as mechanisms to hinder the body’s antiviral response [38]. The viral mechanisms of IFN-I evasion are multifaceted, including sequestering and shielding RNA within double-membrane vesicles, modification of viral mRNA 5′-cap structures, and specific targeting of antiviral cellular pathways [39,40]. In SARS-CoV and MERS-CoV, INF-I production is protective only at the early stages after infection; at later time points, on the contrary, when exaggerating immune response takes place, IFN-I and inflammatory cytokines become pathogenic [41].

No studies on the ability of fenretinide to block INF-I evasion in coronavirus have been reported so far, but, by analogy with Flaviviridae, some fenretinide activity on the mechanisms regulating the INF-I evasion in coronavirus might be expected.

Fenretinide treatment has been found to inhibit HIV infection by perturbing cell membrane structure and inhibiting crucial early events in the HIV fusion process [42]. Fenretinide indeed is known to modify the biosynthesis and metabolism of ceramides, therefore, changing localized membrane domain organization. Cell membrane modifications might alter the trafficking of virions through the endocytic pathway, thus leading to non-productive infection [42,43].

SARS-CoV-2 predominantly infects airway and alveolar epithelial cells, vascular endothelial cells, and macrophages by linking angiotensin-converting enzyme 2 (ACE2) that is abundantly expressed in the human lungs. After linkage to ACE2, the viral entry into the host cells is mainly mediated by endocytosis. Consequently, inhibition of cell entry may be expected by treatment with fenretinide.

It has been observed that the endocytic pathway of SARS-CoV-2 also involves autophagy with the formation of autophagosomes and autolysosomes [44]. Autophagy represents an essential cellular process, decreasing virus infections. Beclin1 (BECN1) is one of its key regulators.

MERS-CoV replication results in reduced BECN 1 levels, with a consequent block of the fusion of autophagosomes and lysosomes [45]. Thus, autophagy-inducing, BECN1-increasing agents may have antiviral effects [46].

Fenretinide has demonstrated autophagy-inducing ability in different cell types. In human mammary carcinoma cell line MCF-7, fenretinide triggered an autophagic cell death pathway mediated by an increase in Beclin 1 expression [47]. In human pancreatic cancer cells, fenretinide induced autophagy by ROS increase [48]. In gastric carcinoma HCG27, autophagy was stimulated by the accumulation of dihydroceramide induced by fenretinide [49]. 

Another target for antiviral activity is the AKT1/mTOR pathway. Indeed, a kinome analysis of MERS-CoV-infected cells has detected phosphorylation changes of several regulatory kinases, including AKT1 and mTOR. These changes have been correlated to autophagy [50,51]. The pharmacological use of AKT1- and mTOR-pathway inhibitors has shown promise for antiviral therapy against several viruses, including MERS-CoV [51]. 

The inhibitory effect of fenretinide in AKT1 and mTOR pathways has been reported in tumor cells [52] and in monocytes/macrophages [27,53]. 

All these findings clearly show the involvement of fenretinide in multiple pathways, controlling viral replication. Therefore, even in the absence of specific studies on SARS-CoV-2, an indirect antiviral activity of fenretinide may be supposed through the induction of an “antiviral environment”, hindering supportive steps of viral replication, both at a cellular level and in the infected tissues.

## 5. Tolerability of Fenretinide

Fenretinide has been well-tolerated in many clinical trials aimed at evaluating its antitumor activity in pediatric and adult cancers. Oral administrations of fenretinide in corn oil gelatin capsules (4000 mg/m^2^ for 28 days) or in a lipid matrix (Lym-x-sorb, 61 mg/kg for 7 days) have been well-tolerated at high doses and for protracted periods of time. No significant hematological alterations or dose-limiting side effects have been obtained [9,54] in these studies. Many other clinical trials with fenretinide have confirmed its tolerability [17,55,56]. 

A phase II study reported thrombocytopenia, anemia, hepatosplenomegaly in patients with myelodysplastic syndromes orally treated with fenretinide at 300 mg/day for 4 weeks and escalated to 400 mg/day for other 8 weeks [57]. Thrombocytopenia was also observed in a phase I study in some patients with hematologic malignancies after administration of fenretinide (905 mg/m^2^) as a continuous intravenous infusion for five consecutive days [58]. In a very recent study in mice, oral fenretinide led to splenomegaly and increased the circulating leukocytes [59].

A trial aimed at evaluating the effect of fenretinide as a chemopreventive agent included 1435 patients that received a 5-year fenretinide oral treatment. Endpoints considered for safety assessment indicated that fenretinide was endowed with good tolerability. Indeed, the most common adverse events were diminished dark adaptation (19.0%) and dermatologic disorders (18.6%). Less common events were gastrointestinal symptoms (13.0%) and disorders of the ocular surface (10.9%). These symptoms occurred during fenretinide treatment and recovered over time [12,60]. 

## 6. Pulmonary Delivery of Fenretinide in COVID-19

In COVID-19 patients, the rapid evolution of the disease requires prompt treatment because only a narrow time window exists for therapeutic intervention. In this regard, pulmonary drug delivery offers the advantage of carrying the bioactive molecule in direct contact with the pathological lung epithelia, thus ensuring a rapid onset of the therapeutic response. High local drug concentrations may be easily obtained by pulmonary administration with a concomitant increase of the pharmacological effect but without the side effects elicited by other administration routes.

Drugs administered by enteral or parenteral routes, indeed, need to reach the blood circulation to be distributed to tissues and organs and enter the pathological site. This general distribution may provide extensive side effects, particularly when high drug administration doses are required to achieve a therapeutically active concentration of the drug in the pathological site.

The increased pharmacological effect provided by high drug local concentrations and the decreased toxicity due to lack of a systemic distribution may improve the overall therapeutic efficiency of the drugs administered by pulmonary route, as demonstrated in several respiratory diseases, such as asthma, cystic fibrosis, and chronic obstructive pulmonary disease [61].

**Figure 2:**
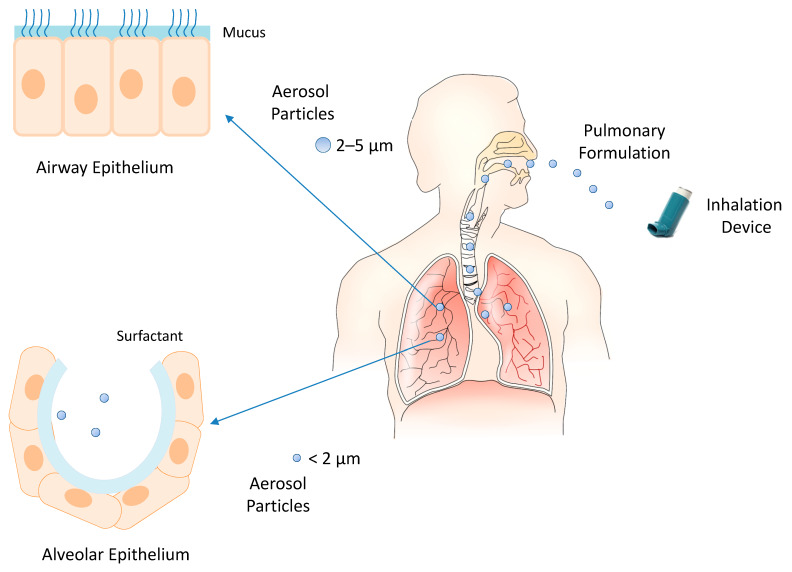
Inhalation of pulmonary formulations and size-dependent distribution of aerosol particles in the respiratory tract.

The inhalation devices play a crucial role in the effectiveness of pulmonary drug administration. The most common devices are nebulizers (e.g., jet nebulizers, ultrasonic nebulizers, and vibrating mesh nebulizers), metered-dose inhalers, and dry powder inhalers [62]. The selection of the inhalation device depends on the physicochemical characteristics of the drug and its formulation. Liquid formulations are administered by nebulizers and metered-dose inhalers, and solid formulations by dry powder inhalers. In each case, the inhalation device provides aerosol particles whose size can control the extent of inhaled drug accumulation and the site of drug deposition within the airways. Smaller particles achieve a greater total drug accumulation in the lungs and farther distal airway penetration compared with larger particles. Particles smaller than 5 μm in diameter may flow in the airstream beyond the retro-pharynx and reach the trachea. Particles of 2–5 μm in diameter are deposited in the upper respiratory tract at the level of the trachea and tracheal bifurcation. Particles smaller than 2 μm in diameter deposit in the lower airway and alveolar epithelia [63,64]. Then, the modulation of intrapulmonary deposition through the control of the aerosol particle size can appreciably improve the inhalation drug therapy (Figure 2). The pulmonary administration of drugs mainly provides a local therapy but may also provide a systemic therapy when the physicochemical characteristics of the drugs can support their absorption through the alveolar epithelium at extents suitable to elicit systemic effects. Indeed, the large surface area, extensive vascularization, and single-cell barrier in the alveoli make the lungs an appropriate portal for the systemic absorption of molecules, such as insulin, human growth hormone, etc. [65].

**Figure 3:**
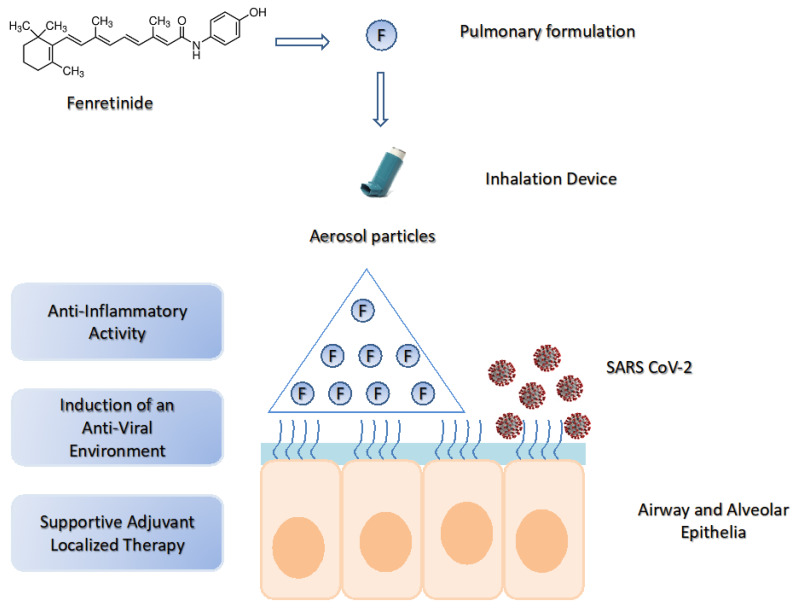
The potential use of fenretinide in COVID-19 by pulmonary delivery. SARS-CoV-2 lung infection triggers excessive inflammation and activation of the cytokine storm. Pulmonary delivery of fenretinide can provide high drug concentrations in the lung airway and alveolar epithelia, thus inducing a rapid onset of anti-inflammatory activity and an “antiviral environment”. This generates a supportive adjuvant localized therapy useful in multimodal treatments.

Pulmonary delivery of fenretinide could be a valuable tool in COVID-19 due to the possibility of obtaining a very high drug concentration in the airway and alveolar epithelia, thus triggering a rapid onset of local anti-inflammatory response. At the same time, the ability of fenretinide to induce an “antiviral environment” could further enhance its therapeutic efficacy (Figure 3). 

In order to be effective, pulmonary fenretinide formulations should provide aerosol particle size smaller than 2 μm, for deposit in the lower airway and alveolar epithelia, where the infection process is amplified by the extensive vascularization. Moreover, after deposition, they should trigger a rapid drug release to speed up the onset of the therapeutic activity.

Such formulations require fenretinide solubilization in an aqueous phase, and the adequate solubilization degree to provide high concentrations of the bioavailable drug, in the lungs, after inhalation. 

Unfortunately, the hydrophobic character of fenretinide strongly hinders its aqueous solubilization. Moreover, the possibility to use solubilizing agents, such as tensides or water-mixable co-solvents, is severely restricted, by tolerability issues, in the formulations destined to inflamed lungs.

Highly tolerated, aqueous fenretinide formulations have been obtained by complexation with cyclodextrins [52] or encapsulation in nanomicelles [66]. 

Complexation with 2-hydroxypropyl beta-cyclodextrin has increased fenretinide aqueous solubility from 0.017 mg/mL (pure drug) to 2.41 mg/mL (complex). The aqueous formulation of the complexed drug, administered by the parenteral route, was well-tolerated and increased the drug bioavailability and antitumor activity in mouse models of different tumor types [52].

Nanoencapsulation in phosphatidylcholine-glyceryltributyrate nanomicelles has increased fenretinide’s aqueous solubilization up to 3.88 mg/mL (nanoencapsulated drug). The intravenous administration of the nanomicelles in mice bearing tumor xenografts showed enhanced drug bioavailability and antitumor activity [66]. Moreover, high tolerability was demonstrated by the absence of adverse effects after repeated administrations and for protracted periods of time. 

Therefore, the in vivo tolerability and the ability to provide high fenretinide solubilization levels suggest that complexation with cyclodextrins [52] or encapsulation in nanomicelles [66] can be valuable means for preparation of safe and efficient pulmonary fenretinide formulations.

## 7. Drugs Currently Used in COVID-19

There is presently no vaccine or documented specific, standard treatment against COVID-19. Most of the potential drugs are being investigated for safety and efficacy in SARS-CoV-2 infection, but, until now, no drugs are validated to have significant efficacy in the clinical treatment of COVID-19 patients in large-scale studies. Current treatments are using drugs that were originally designed for other pathologies and have been repurposed for COVID-19 trials. 

These include antiviral and immunomodulating drugs designed to boost the innate immune response or to inhibit the inflammatory processes, causing lung injury. Drugs for symptomatic control are also employed.

### 7.1. Antiviral Drugs

Antiviral drugs act on the coronavirus by direct mechanisms, inhibiting key viral enzymes responsible for genome replication or hindering viral entry to human cells. 

Remdesivir is the most promising, among the antiviral drugs. It exhibits broad-spectrum antiviral activity against RNA viruses. In previous studies, it showed antiviral activities, both in vitro and in vivo, against different coronaviruses, including SARS-CoV and MERS-CoV [67,68]. In a recent study, remdesivir inhibited SARS-CoV-2 in vivo [69].

Ivermectin is an anti-parasitic agent, which has proved to exert antiviral activities toward both HIV and dengue virus [70]. Recently, an in vivo study put in evidence its capability to reduce viral RNA up to 5000 fold in SARS-CoV-2 infection [71].

Lopinavir/Ritonavir are protease inhibitors. They are used in combination in the treatment of patients with HIV infection. Several clinical, preclinical, and in vitro studies performed on SARS-CoV and MERS-CoV proved that the lopinavir/ritonavir combination was effective against these viruses [72,73,74].

Hydroxychloroquine is active against malaria as well as autoimmune diseases (such as rheumatoid arthritis, lupus erythematosus). It has been recently investigated as a potential broad-spectrum antiviral drug for its ability to increase the endosomal pH, which prevents virus-cell fusion [75]. Hydroxychloroquine has been shown to specifically inhibit the replication of SARS-CoV by interfering with the glycosylation of its cellular receptor ACE2 [76]. Recent in vitro studies revealed its ability to effectively reduce the viral copy number of SARS-CoV-2 [77].

### 7.2. Immunomodulating Drugs Enhancing the Innate Immune System

Immunomodulating drugs able to stimulate innate antiviral immune responses are now repurposed for the treatment of COVID-19. Natural Killer (NK) cells represent a highly specialized lymphoid population of the innate immune system with potent activity against virus-infected cells. 

Migration of NK cells and macrophages to the lungs has been demonstrated to play a major role in the clearance of SARS-CoV [78]. Indeed, the innate response itself can control SARS-CoV infection, without the help of CTLs and antibodies, particularly in the early stages of the infection process. 

Type I interferons are secreted by virus-infected cells and represent a host protective mechanism. Recombinant interferons, used alone or in combination with other drugs, have provided a broad-spectrum antiviral activity against HCV, respiratory syncytial virus, SARS-CoV [79], and MERS-CoV [73]. Recent trials are assessing their safety and efficacy in COVID-19. 

### 7.3. Immunomodulating Drugs Attenuating the Inflammatory Response

Drugs interfering with the IL-6 pathway are used to attenuate the inflammatory response in COVID-19 [80]. Elevated IL-6 levels are predictive of poor prognosis in patients with ARDS [81]. The pathway of IL-6 signaling occurs through IL-6 receptors, which are expressed predominantly on neutrophils, monocytes, macrophages, and some lymphocytes [82]. Tocilizumab and siltuximab are monoclonal antibodies against IL-6. TZLS-501 is an anti-IL-6 receptor antibody, and sarilumab an IL-6 receptor antagonist. They all are designed to inhibit the binding of interleukin-6 to its receptors, thus alleviating cytokine release syndrome.

Thalidomide has been repurposed for the treatment of COVID-19 due to its anti-angiogenic, anti-inflammatory, and anti-fibrotic activity. Its anti-inflammatory activity is mainly based on the inhibition of TNF-α production. Thalidomide has shown promise in the treatment of multiple inflammatory diseases, such as lupus erythematosus and Crohn disease [83]. Preclinical studies proved that thalidomide was effective in treating mice infected with influenza virus H1N. It decreased the infiltration of inflammatory cells and inhibited the production of inflammatory cytokines [84]. Recent studies are evaluating its efficacy in SARS-CoV-2 [85].

Lenalidomide is a derivative of thalidomide. It is currently used in the maintenance therapy of multiple myeloma due to its immunostimulatory effect on NK cell number and function combined with an acceptable toxicity profile. Lenalidomide has been proven to limit the amount of pro-inflammatory cytokines, such as TNF-α, IL-12, IL-1, IL-6, and increased IL-2 and IFNγ [86,87]. Many studies have demonstrated the lenalidomide’s strong anti-angiogenic activity [88,89,90,91]. With respect to thalidomide, lenalidomide is expected to provide a superior activity and decreased side effects in COVID-19 treatment.

Glucocorticoids have been used in SARS-CoV and MERS-CoV infections to restrain lung inflammation and immune responses [92,93,94]. Their use has provided side effects, such as secondary bacterial infections, osteoporosis, and prolonged viral clearance. Glucocorticoids, such as methylprednisolone, are now under evaluation in COVID-19 for effectiveness and safety [85].

## 8. Possible Fenretinide Combinations with Drugs Currently Used in COVID-19

Pulmonary administration of fenretinide, in combination with the drugs currently used in COVID-19 treatment, could represent a new, effective tool to improve the efficacy of the single drugs and produce a strengthened overall pharmacological response.

Fenretinide is particularly suitable to be used in combination because its poly-pharmacology may reinforce the activity of several drugs, and its high tolerability may enable large adjustments of the administered dose without toxicity concerns.

The combination of fenretinide with the antiviral drugs used in COVID-19 is expected to improve the treatment efficacy due to the ability of fenretinide to induce an “antiviral environment” that can behave as an adjuvant to the specific antiviral activity of the drugs.

In combination with the immunomodulating drugs, fenretinide may contribute to enhance the innate immune response due to its stimulating effect on NK cells’ proliferation and cytotoxicity [95,96]. Moreover, its anti-inflammatory activity, largely demonstrated in several models, may boost the attenuation of the inflammatory response induced by the immunomodulating drugs. In particular, the ability of fenretinide to decrease IL-6 [25,27] should improve the efficacy of the drugs blocking the IL-6 pathway, such as tocilizumab, siltuximab, sarilumab, TZLS-501.

The combinations of thalidomide or lenalidomide with fenretinide are expected to be very effective due to the multiplicity of mechanisms involved in the anti-inflammatory activity of both types of drugs. Particularly, the decrease of TNF-α [25,26] and the antiangiogenic effect [97,98] provided by fenretinide, added to the same effects provided by thalidomide and lenalidomide, should boost the anti-inflammatory response elicited by the combination. Fenretinide and lenalidomide were evaluated, by intravenous administration, in a mouse model of neuroblastoma. High tolerability and antitumor activity were obtained with this combination [66]. 

In regard to the administration route, the drugs to combine with the pulmonary fenretinide are expected to be administered by enteral or parenteral routes, depending on the form of the formulations available on the market. However, the pulmonary administration route would be the best option because, as already reported for fenretinide, the local drug administration in the pathological site would provide a rapid onset of the therapeutic response. Moreover, in addition to local therapy, systemic therapy may also be obtained if the drug absorption through the alveolar epithelium can take place at a suitable extent.

## 9. Conclusions

The utility of fenretinide anti-inflammatory activity, associated with its high tolerability, has been repeatedly demonstrated in lung-related pathologies, such as cystic fibrosis, allergic asthma, and chronic lung infections. Its antiviral activity in the zika virus, dengue virus, respiratory syncytial virus, hepatitis C virus, and HIV has been largely proved and attributed to several mechanisms of action. Although there is no direct evidence of fenretinide application in COVID-19, the multiple inhibition mechanisms demonstrated in the studied viruses and the involvement of fenretinide in multiple pathways controlling viral replication strongly suggest that fenretinide could provide an indirect antiviral activity towards SARS-CoV-2, as well as other virus types, by means of the induction of an “antiviral environment” hindering infection. Therefore, both the anti-inflammatory activity and the ability to induce an “antiviral-environment” indicate that fenretinide may have supportive adjuvant utility in treating COVID-19. Fenretinide administration by means of pulmonary formulations may further improve its therapeutic value by providing high drug concentrations in the pathological lung epithelia and a fast onset of the drug activity, which is particularly important in SARS-CoV-2 infection, where only a narrow time window exists for therapeutic intervention. Moreover, the pulmonary administration of fenretinide, in combination with the drugs that are currently used in SARS-CoV-2 infection, could represent a new, effective tool in COVID-19 treatment.
